# Value-Based Healthcare Project Implementation in a Hierarchical Tertiary Hospital: Lessons Learned

**DOI:** 10.3389/fpubh.2021.755166

**Published:** 2022-02-03

**Authors:** Carolina Varela-Rodríguez, Albert García-Casanovas, Blanca Baselga-Penalva, Pedro M. Ruiz-López

**Affiliations:** ^1^Quality of Care Unit, Hospital Universitario 12 de Octubre, Madrid, Spain; ^2^Instituto de Investigación Biomédica del Hospital Universitario 12 de Octubre I+12, Madrid, Spain; ^3^IMIM (Hospital del Mar Medical Research Institute), Barcelona, Spain

**Keywords:** value-based healthcare, operative implementation, quality of care, resources estimation, patient- reported outcomes (PRO), patient-reported experience (PRE), clinician-reported outcomes (CRO)

## Abstract

An important innovation in healthcare is the value-based healthcare (VBHC) framework, a way to solve health services' sustainability problems and ensure continuous improvement of healthcare quality. The Quality and Safety Unit at the Hospital Universitario 12 de Octubre has been since May 2018 coordinating the implementation of several healthcare innovation projects within the paradigm of VBHC. Implementing innovations in a complex institution, such as a tertiary hospital, is a challenge; we present here the lessons learned in the last 4 years of work. We detail exclusively the aspects related to continuous improvement and value addition to the process. In summary, for any VBHC project implementation, we found that there are five main issues: (1) adequate data quality; (2) development of data recording and visualization tools; (3) minimizing healthcare professional's effort to record data; (4) centralize governance, coordination, and transparency policies; (5) managerial's implication and follow-up. We described six steps key to ensure a successful implementation which are the following: testing the feasibility and complexities of the entry process; establishing leadership and coordination of the project; developing patient-reported outcomes and experience measurements; developing and adapting the data recording and data analysis tools; piloting in one or more medical conditions and evaluating the results and project management. The implementation duration can vary depending on the complexity of the Medical Condition Clinical Process and Patient Pathways. However, we estimate that the implementing phase will last a minimum of 18 and a maximum of 24 months. During this period, the institution should be capable of designing and implementing the proposed innovations. The implementation costs vary as well depending on the complexity, ranging from 90,000 euros to 250,000 euros. Implementation problems included the resistance to change of institutions and professionals. To date, there are few successful, published implementations of value-based healthcare. Our quality of care and patient safety methodological approach to the implementation has provided a particular advantage.

## Introduction

Offering value-based healthcare is a tempting opportunity for any healthcare institution (1–6), and, to do so, a systematic measurement of health outcomes is the necessary first step for any healthcare process evaluation and improvement (2, 7, 8). Moreover, any real innovation of the healthcare process must favor sustainability and equity, be very adaptive to a dynamic environment and ensure the best possible care in any circumstance (including crises such as pandemics) (9–11).

**Figure 1:**
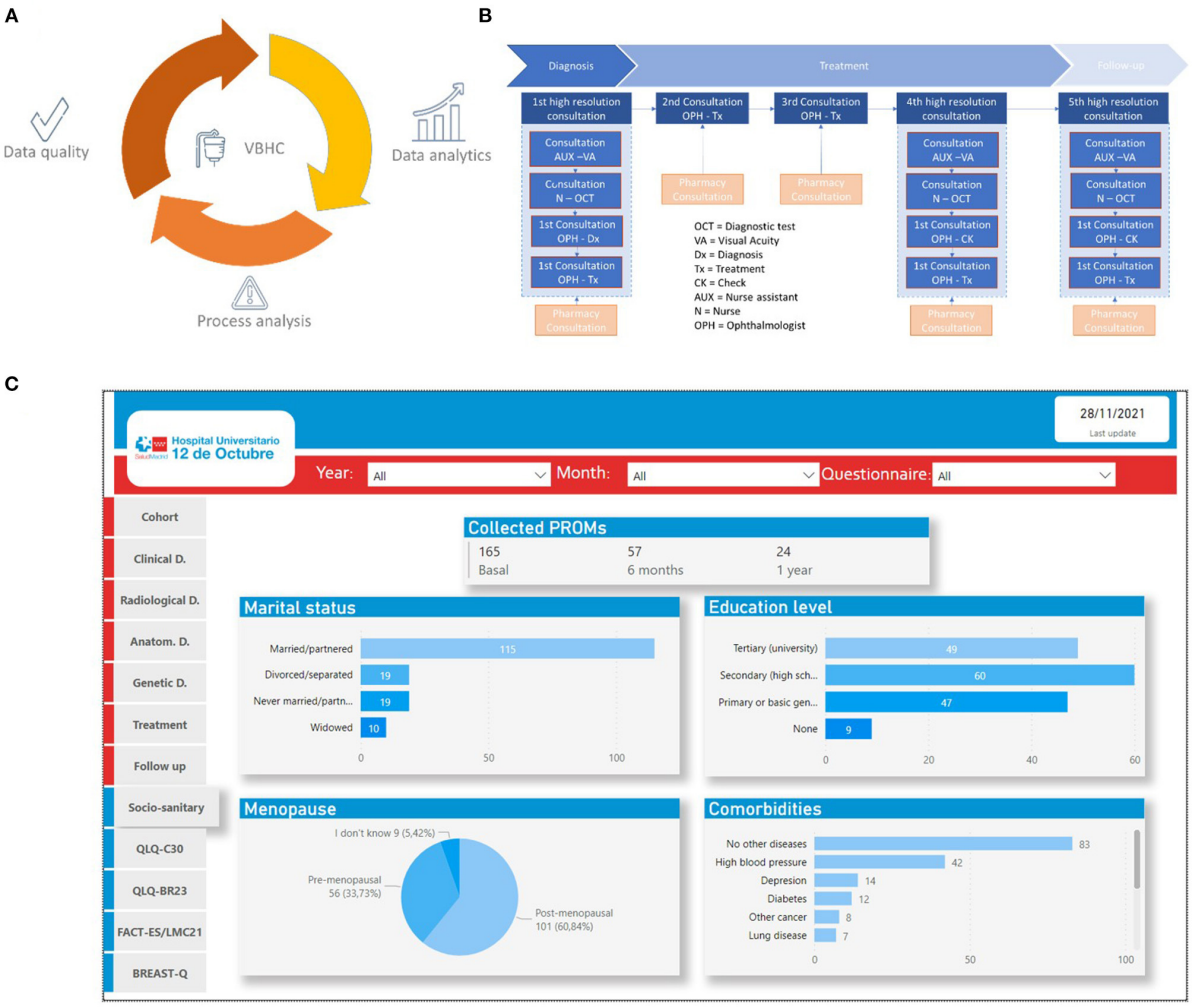
**(A)** Value-based healthcare (VBHC) requires good data quality, deep knowledge of the medical condition process, and meaningful data analysis. Data recording tools have to ensure easy data recording, appropriate data quality, and confidentiality of the information. The clinical process analysis will help identify how to adapt tools and ensure appropriate quality and safety of the process and data, defining the primary source for each variable measured, the moment to record it, and how this is done within the medical condition process. The epidemiological analysis tools should grant “real-time” methods and quick feedback to the patient care team. **(B)** Example of ADMD clinical process analysis; **(C)** Example of the dashboard from the breast cancer cohort.

Value-based healthcare (VBHC) is an international trend that implies significant changes at several levels of the healthcare institutions from managerial viewpoints to the doctor-patient relationship (1, 12). Therefore, implementing and evaluating these innovations needs some structure and considerable effort (1, 13). As an institutional strategy for continuous improvement of healthcare quality, implementing systems to measure value for patients, populations, and professionals was essential, along with organizing the healthcare practice around clinical processes (medical conditions) instead of specialties, services, or units (Figure 1). Moreover, to calculate the value, it is necessary to measure the costs *per patient* through the entire process (12, 14). Since May 2018, the Quality of Care and Patient Safety Unit at the Hospital Universitario 12 de Octubre (HU12O) has been coordinating the implementation of five healthcare innovation projects (Supplementary Table S1), including the following clinical conditions: lung cancer (LC), age-related macular degeneration (ARMD), inflammatory Bowel Disease (IBD), breast cancer (BC), and coronavirus disease (COVID). Those projects are currently at different stages of implementation and imply different complexity and resource allocation.

The hospital is also part of a European Consortium of Hospitals called VOICE for breast and lung cancer outcomes research coordinated by the Institute for Health Services Research Kronikgune. We are part of the Spanish Consortium of Hospitals EIIMPROVE for IBD, and in a Spanish Community of Hospitals in ARMD sponsored by Novartis.

As in any complex and learning organization, any medical act is as much a source of data and information as it uses data and information from past medical acts transformed in knowledge through a shared reflection. Therefore, the data quality of the variables is key to allowing proper healthcare and avoiding errors and biases. Recording from the primary source, either the clinician (clinician-reported outcomes measures; CROM) or the patient (patient-reported outcomes measures; PROM) variables, reduces the variability of data quality. The effort of translating data to clinical decision aids benefits from an appropriate data visualization and a methodologically robust analysis (Figure 1C).

Contexts are quite different among different institutions and countries. Our experience is limited, but a lot of the work already done could be adapted to other cases to help avoid mistakes. We present in this work the strategies and approaches that assist us in the implementation and the barriers identified.

## Materials and Methods

### Organizational Setting and Context

The population attended by the Hospital Universitario 12 de Octubre, and the inclusion criteria for each Cohort were the ones defined by the Communities adapted to the characteristics of the hospital. The inclusion criteria are detailed in the Supplementary Material. Several study designs were used during the implementation of the VBHC innovation. From qualitative studies to prospective cohort studies. The protocols are available in Spanish if needed, and all were approved by the Ethics Committee when required. The approval documents are available as well in Spanish. The consensus studies were either focal and discussion groups with key stakeholders or modified Delphi studies with experts. During the implementation process, the piloting study was a descriptive observational cohort study.

### Process Definition and Limits

Our projects focus on the in-hospital part of the complete medical condition process. However, the process was analyzed from the initial diagnosis (suspected diagnosis) in primary care or screening program to the last follow-up consultation in either primary care or hospital care.

The process analysis included the following:

- Workflow
- Archetype patient journey covering at least 80% of the causes of each medical condition
- Identification of the variables of interest within the process workflow
- Information flow from the data generation to the warehousing of the data
- Identification data life cycle within the process: primary sources of the data; databases, communication within the databases, and the person or machine responsible for the data generation and recording

### Variables

The clinical variables included in the standard dataset (ICHOM data set for breast cancer, lung cancer, ARDM, and IBD) and some variables chosen by the physicians and nurses *ad hoc* in every project, patient-reported variables from the PROM and patient-reported experience measure (PREM) questionnaires, variables of process indicators, (e.g., times and delays, a numerical account of activity and outputs, costs information).

### Hardware

The server must have at least RAM with 4 GB, four cores in the central processing unit (CPU), a 64 bits operative system, and a minimum storage space of 15 GB. The computers must have RAM with 9 GB, 64 bits operative system, and four cores in the CPU. Patients should have access to the Internet by computer, smartphone, or tablet for PROM and PREM recording.

### Software

- Tools for data recording that warrants confidentiality and data quality: Redcap (Vanderbilt, USA) or similar solutions. Institution Electronic Health Record system (HCIS) (Madrid, Spain) as CROM source and recording and PROM/PREM recording platforms.
- Statistical analysis software, the amount of data to be managed is going to grow exponentially and therefore is necessary to have professional statistical software to analyze the data. We have used the R package, an open-source software.
- Visualization software to construct dashboards for patients and cohort follow-ups, such as Power BI (Albuquerque, Nuevo México, USA) software or *ad hoc* tools as HOPES (Valencia, Spain) adapted and developed by IDIEIKON (Valencia, Spain) for CROM and PROM or PanelHealth (Madrid, Spain) for PREM.
- ICT Software for project management, coordination, and team communication: Microsoft TEAMS (Albuquerque, Nuevo México, USA) and Google Drive (Perth, Australia), Dropbox (San Francisco, California, USA), Miro, Trello (New York, New York, USA), and Slack (Vancouver, Canada).
- Internal and external communication: Microsoft Office (Albuquerque, Nuevo México, USA), Slidesgo (Malaga, Spain), Powtoon (Londres, United Kingdom), Piktochart, Canva (Perth, Australia), and Pixabay (Berlin, Germany).

## Results

### Patient Recruitment

At the cut-off time for the Cohort in August 2021 for the breast cancer project, 148 patients were included in the Cohort with an average age of 56 years. One thirty-six (91.9%) of the patients agreed and filled up the baseline PROM, and all the 57 participants with 6 months follow-up filled in the 6-month questionnaire. The first 24 patients within the Cohort were followed for 1 year and filled in the 1-year follow-up questionnaire. For the lung cancer project, 110 patients were included in the Cohort and had an average age of 69 years. In this study, 98 (89.1%) of the patients agreed to fill out the baseline PROM. No patient has yet been followed for 6 months. In ARMD, patients are currently being recruited, and in IBD, the tools are still being adapted. During the 3 years, four projects were conducted with more than 200 patients included in the cohorts. Two of our physicians have collaborated in the expert panel for the COVID-19 ICHOM dataset and partially implemented it in the last year. The five projects before the piloting had engaged more than 70 professionals and around 30 patients for tools design and adaptation.

### Working Teams and Responsibilities

The workload was arranged in five teams with specific objectives for the implementation and skills of the team member, the managerial team (MT) focused on project advocacy and coordination; the technological team (TT) focused on technological integration and coordination with external technological partners; the cost-analysis team (CAT) focused on cost analysis and economic evaluation; the clinical team (CT) focused on process analysis, database and dataset agreement, and clinical interpretation of results. Finally, the data analytics and research team (DART) focused on quality of life and experience analysis. The average composition of the teams was 5–8 professionals.

### Improvement Cycle

More than 20 improvement actions have been identified. Three have been prioritized regarding the waiting time for chemotherapy, the image, and the patient information, clinical and organizational.

### Resources and Budget

From our experience, we have successfully applied for private funding for the projects, and we estimated the implementation costs. We assumed two main cost groups, one derived from technological needs (hardware and software) and the second being human resources. As explained, human resources are classified as essential if their roles are compulsory for the project or advisable if they help improve the quality of the implementation, methods robustness, or quicken the process. Thus, we considered a total budget with costs derived from essential and advisable profiles participation the total budget; and an adjusted budget excluding the advisable profiles and including the essential ones exclusively. Using an *ad hoc* complexity assessment, we classified projects in low, medium, and high complexity since they implied different costs, due mainly to more or less human resources required.

From the total budget, hardware and software entailed 30.3% and human resources the 69.7%; in the adjusted budget, the technological resources consumed 35.6% of the total and human resources a 65.4%. The detailed weight was as follows. Eighty-six percentage (around 75,000 euros) of the technology investment was dedicated to software development (recording storage and data visualization platforms, database integration, dashboard design, and coding), and 14% (around 12,000 euros) was used to buy hardware and data analytics software.

Table 1 shows the internal implementation costs; therefore, the horizon time for this budget is 18 to 24 months; the time estimated for implementing the innovation according to the complexity of the different medical conditions considered.

**Table 1:** Budget impact of each professional role key for implementation.

**Human resources**	**E**	**A**	**Person *per* year (Average estimated)**	% Total HR (**)	% Adjusted HR (**)	% Total budget (**)	% Adjusted budget (**)
Medical condition leaders	X		1	29.07%	38.76%	20.10%	24.31%
Managerial leader		X	0.1	3.20%	0.00%	2.21%	0.00%
Communication manager	X		0.1	2.03%	2.71%	1.41%	1.70%
Project manager	X		0.5	10.17%	13.57%	7.04%	8.51%
Quality and Safety coordinator (15)	X		0.25	6.54%	8.72%	4.52%	5.47%
Process engineer or analyst		X	0.25	6.54%	8.72%	4.52%	5.47%
Data manager		X	0.75	10.90%	0.00%	7.54%	0.00%
Epidemiologist/data scientist	X		0.5	10.17%	13.57%	7.04%	8.51%
Case manager		X	0.75	10.90%	0.00%	7.54%	0.00%
ICT engineer	X		0.2	5.23%	6.98%	3.62%	4.38%
EHR referral	X		0.2	5.23%	6.98%	3.62%	4.38%

E, Essential (a must-have for the project); A, Advisable (needed for an optimal implementation). (*) Salaries calculated within the Spanish socioeconomic context. (**) Estimation for medium complexity projects. The complexity classification was developed with a Delphi study.

## Recommendations

### Implementation Process *per* Medical Condition

The implementation process had a duration that varied depending on the complexity of the Medical Condition. However, we estimate that the implementation phase will last a minimum of 18 and 24 months, depending on the medical condition's clinical process complexity.

For the sake of clarity, we have divided the timeline into semesters, from one to four. The first semester is the moment for inclusion of the medical condition in the implementation procedure, analysis of the situation, resources estimation, and advocacy of the project within the CT, complexity, and feasibility evaluated. The tools for proper data recording and teams' coordination should be implemented during these months.

The following two semesters are the piloting phase that will help test the tools and evaluate the appropriateness of the innovation applied in this particular medical condition. During the four-semester, the institution has to introduce the innovations within the daily tasks of the clinical process (with minimum intervention of the project and data managers), to analyze the first-year data, and give feedback to the clinicians and patients with the evaluation of the health technology innovation proposed (DART and CAT). After that, the innovations should work out in the daily care process without incrementing the professionals' workload. However, there are at least 6 months for adaptation to the daily work.

**Figure 2:**
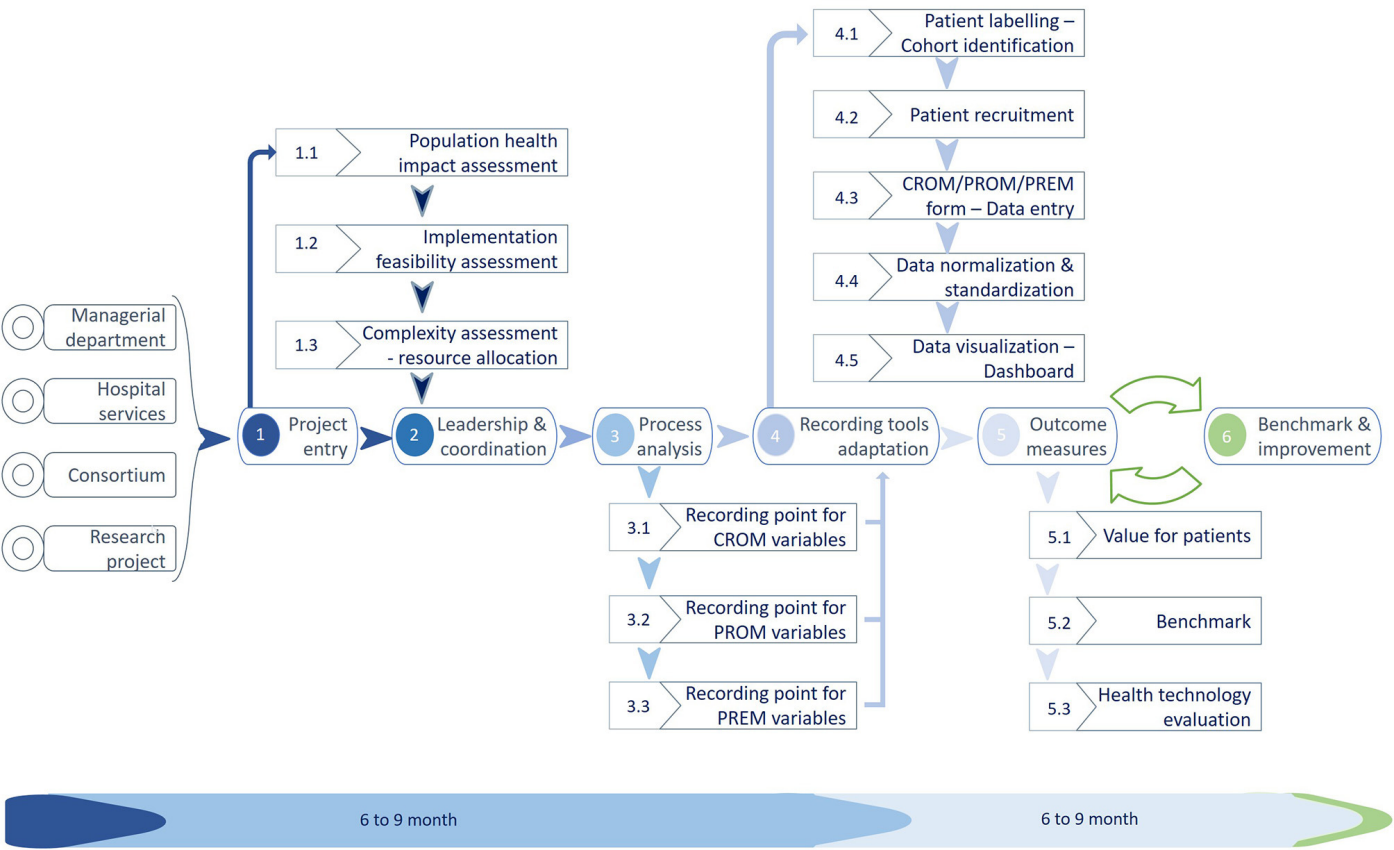
Implementation of the VBHC project process. During the first 12 months, approximately the institution should have settled the tools necessary to begin the data recording with enough quality routinely in the daily work (Icons credits: www.slidesgo.com).

Figure 2 represents the proposed implementation process from the initial idea for a given medical condition until the end of the implementation. From there on, the implemented innovation should become part of daily healthcare.

#### Instructions for Use: Process Steps

1. PROJECT ENTRY: The proposal to include a new medical condition usually came from the hospital services or the managerial department, a consortium such as ICHOM, or a research project. Once proposed, the coordination team evaluates the population (9, 16, 17) and patient health impact (appropriateness of the innovation), and the feasibility of this particular project (feasibility of the project) in this particular moment of the institution. Once considered appropriate and feasible, the institution needs to estimate the resource allocation to make it available (complexity assessment). To do so, we developed two tools for complexity and feasibility assessment.
2. LEADERSHIP AND COORDINATION: The project coordinator usually manages more than one project and depends on the MT. It is advisable and normally done either by the Quality-of-Care Units or Innovation Units since the professionals have the necessary skills. Identifying the clinical leaders within the medical condition will be the next step. Both the MT and the project manager will be in charge of the task.
3. PROCESS ANALYSIS: Then the implementation team (including CT, CAT, and DART) has to focus on the variables and indicators validation, given healthcare coherence and utility, and once the specialist agrees, it is important to decide on adequate tools for data recording. The clinical process underlying any medical condition helps understand where the health information system data should be recorded and how and who is responsible (primary source) for the key data recording.
4. RECORDING TOOL ADAPTATION: Once consensus is obtained in what, by whom, and when each variable is recorded, the existing tools must be appropriately adapted for the task by the TT. Suppose there is no infrastructure for data recording within the institution. In that case, some external tools can be used and have to be integrated into the Health Information System (HIS) (e.g., PROM or PREM recording platforms). To the best of our experience, that is the trickiest part of the implementation. To collect clinical data, we developed specific forms within the electronic health record system to facilitate data recording by healthcare professionals. For the collection of PROM, the company HOPES has developed the technological infrastructure, and the company Whykers has developed the PREM and the recording tool PanelHealth.
5. OUTCOME MEASUREMENT (PILOT): No innovation is good or bad by design. It has to be evaluated and adapted for each case of use; therefore, after the adaptation and putting in place of tools and the Cohort's follow-up, a piloting phase offers the information to evaluate the innovation benefits, pitfalls, and issues.
6. BENCHMARK AND IMPROVEMENT: If the evaluation proves positive in terms of added benefits to the system and value to the patient, population, and professionals, it must be transferred from innovation to daily work and enter the cycle of continuous improvement.

#### Health Information System: Requisites, Adaptations, and Evolution

Data comes from several primary sources, clinicians, patients, and analytic software (such as laboratory information systems and cost information systems). All data were included in a central repository and shared with the DART and CT to fulfill their primary (health care of individual patients) and secondary (observational studies) objectives.

Once CT has agreed upon the dataset (CROM, PROM, and PREM), these variables are converted in a structured form for data recording and integrated with a system that allows data extraction and sharing. The next step is to enable cohort identification, labeling each patient individually in the Electronic Health Record (EHR). Thus, we can access the individual patient data to export and construct the indicators. Underneath the form, the data (and information) normalization system according to international standards (SNOMED, LOINC, ICD-10…) is autonomous from the professional intervention. This standardization would be the basis for comparison with other organizations.

**Figure 3:**
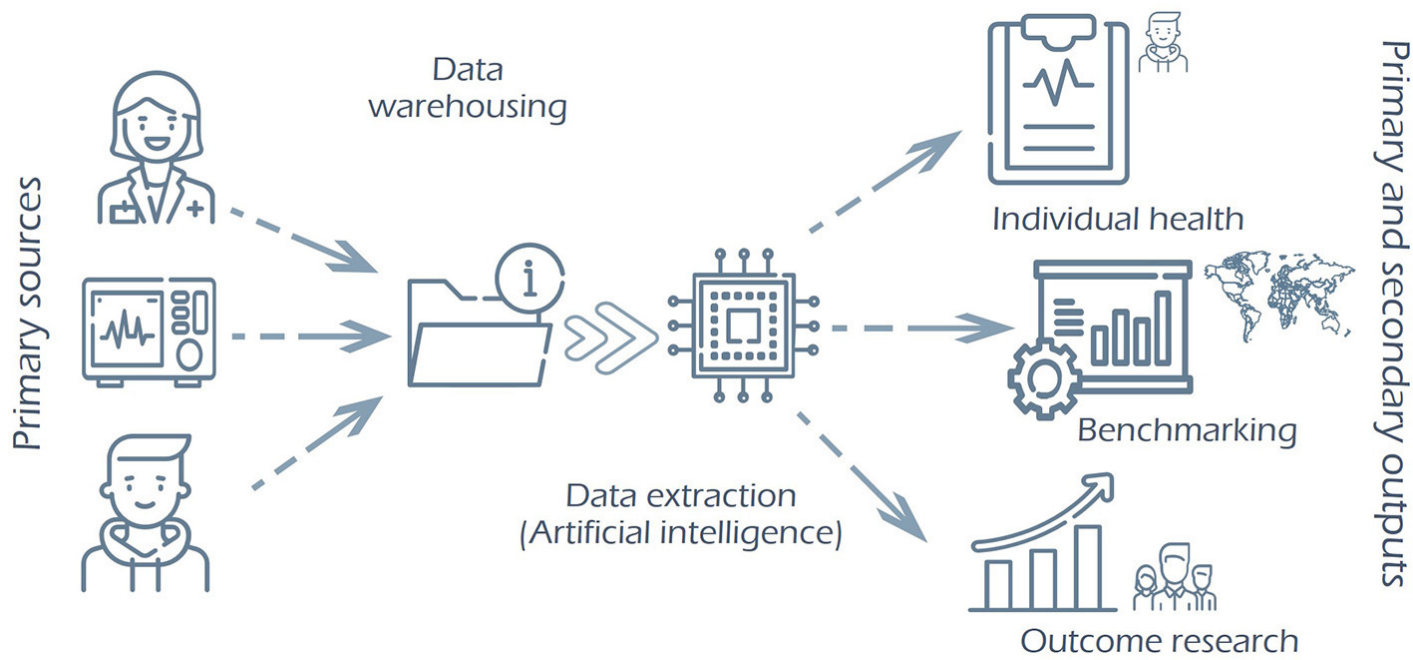
Expected evolution of the Health Information System (HIS). From the primary sources (clinician, diagnose or treatment machines, patients), data is warehoused in a central repository of information, normalized and standardized within an international standard. Thus, the data inside is shareable for multiple purposes, such as patient follow-up, benchmarking, or outcome research.

The expected result is to have a new approach to data recording and availability for primary and secondary use of the information to improve the system and the health results (Figure 3).

#### Anticipated Results

The anticipated results for the HIS and the process improvement are as follow:

1. Versatile and diverse data exploitation (outputs).
2. Usable tools for both clinical practice and research without the need for double data recording.
3. Patients, provided with a forum to talk regularly and systematically with professionals and researchers
4. No information from the EHR is missing. It is advisable to enrich results with the exploitation of unstructured data.
5. Clear data-management governance.
6. Assured the real portability of the data.
7. Reduce unnecessary variability with the continuous improvement cycle based on real-world quality data.
8. Ensures comparability using common recording and analysis methods.
9. Systematic analysis of the data quality
10. Systematic generation of clinical dashboards (Figure 1C) and other decision aids for clinicians.

### New Interdisciplinary Professional Roles

Enrichment of the teams with interdisciplinary and science diversity is provided by hiring new professional specialties (bioengineers, epidemiologists, data analysts and data scientists, data managers, communication and negotiation experts, etc.) and by searching for mixed profiles capable of peer communication among disciplines, e.g., health professionals with a deep knowledge of artificial intelligence and engineers with a high understanding of the clinical process. Table 2 resumes the professional profiles necessary for VBHC implementation. The common vocabulary and concepts make these mixed roles the perfect medium for peer communication and reducing the information gap or asymmetry between team members.

**Table 2:** Professional profiles and competencies, skills and responsibilities; E, essential; A, advisable.

	**E**	**A**	**Skills and competencies**	**Tasks and responsibilities**	**Timing**
Leaders in the medical condition management	X		Highly specialized clinical knowledge in medical conditions. Interpersonal relationships and negotiation skills Communication	Project advocacy and internal communication within the team. Project results presentation in science forums	Whole implementation project
Managerial leader		X	Healthcare management Project management Negotiation and conflict solving skills Empathy and compromise	Project advocacy internally and externally, especially in managerial forums. Professional incentive program Projects performance follow up	The whole first year
Communication manager	X		Internal and external communication skills Persuasion	Diffusion and communication of the project and its milestones to the main stakeholders	The whole duration of the implementation
Project manager (implementation coordinator)	X		Project management Healthcare management Negotiation and conflict solving skills Empathy and compromise Data analytics understanding Process analysis skills	Coordinate the different teams to the same objectives Management and meeting optimization. Project milestones, deliverables, and schedule follow-up Document the project	The whole duration of the implementation
Quality and safety coordinator	X		Process analysis skills Coordination and negotiation skills Communication skills	Process analysis Patient archetype definition and identification Internal coordination	The whole duration of the implementation
Process engineer or analyst		X	Process analysis skills	Analyze the process Redesign the process (continuous improvement)	First semester
Data manager		X	Data Quality knowledge Data's Life cycle understanding	Patient cohort follow-up Request for the proper fulfillment of the variables	Second and third semester
Epidemiologist/data scientist	X		Analysis and data visualization	Design of the analysis and visualization tools. Analysis plan and interpretation of the results	Third and four-semester
Case manager		X	Clinical and care knowledge	Follow-up patients and attend to their care needs	Whole duration
ICT engineer	X		Data life cycle understanding Data interoperability and integration of databases ICT tools design and integration	Database integration Data models and archetypes definition Dataset codification and translation to different standardized models	First and second semester
EHR referral	X		Knowledge of the EHR management EHR modification of the software	Local adaptation of the need into the tools in the EHR	The whole first year

### Implementation Maturity Set of Indicators

A set of indicators of implementation maturity have been developed; however, they have not yet been tested. The proposed set of indicators is as follow (Supplementary Table S3–Supplementary Material):

#### Implementation Process Quality

- Team diversity: Number of different knowledge areas included
- Professional engagement: Number of persons participating in the meeting x 100/persons invited to participate
- Patient engagement: Number of patients that fill up at least one PREM. The number of patients that fulfill at least one PREM x 100/Total patient on the Cohort.
- Performance of the continuous improvement cycle: Number of agreed compromises agreed in teams meetings x 100/total of improvements developed

#### Data Recording Quality

- Recruitment success: Number of patients included in the cohort x 100/total patients with inclusion criteria.
- First intention data fulfillment: Number of data recorded without data manager intervention x 100/all the data that should have been recorded.
- Data recording automation: Number of variables fulfill automatically per patient HER x 100/Total variables from the dataset (CROM).
- Availability of the cost information: Costs available per patient/total costs considered in the cost dataset.

#### Data Quality and Patient Follow-Up

- PROM Recurrence ratio: Number of patients that answer the previous PROM x 100/patients that answer the following PROM
- Follow-up calls: Number of follow-up calls made by the data manager both to patients and professionals

#### Feedback and Improvement

- PROM utilization: Number of professionals access to the PROM x 100/total of professionals caring for the patients.
- Areas of improvement detection: Number of alerts identified
- Alerts response: Number of responses to an alert (e.g., schedule a new appointment with mental health once depression or anxiety levels rise in PROM) x 100/Total number of alerts
- Continuous improvement opportunities: Number of improvements derived from PREM and PROM analysis

#### Managerial Implication

- Professional substitution: Days without a professional role (e.g., case manager) x 100/total of the professional leave days

## Discussion: The 10 Lessons Learned

**ONE:** The main lesson is that implementing VBHC is not free of implementation costs, which means an investment of resources. In agreement with the results from Ackerman et al. (18), we found that a minimum amount of 90.000 euros was required to implement VBHC in medium to high complex medical conditions processes. The institution has to provide the human resources to coordinate, manage and communicate the project. It is important to implement and develop agile tools to understand the healthcare clinical management processes and the outcomes of each medical condition to patients and caretakers. It should facilitate the means to understand the experience of the patients and their families along the healthcare process. It is important to have the human resources to develop and adapt the current Information and Communication Technologies (ICT) tools for the best quality data recording and exploitation in real-time to influence the cycle of the decision-making process with the patients.

**Two:** In the process of data appropriateness and data-recording tools adaptation for outcome measures within the local system, 6 months were spent for the first medical condition to be considered. During the second semester, a key milestone happened when the data of the first 100 patients were collected with the data manager's help. This information will first evaluate the innovation impact and appropriateness (project pilot), and the main technical problems will be identified and solved.

**Three:** The clinical process is the main structure of the healthcare assistance and the appropriated outcome measurement. Skills and knowledge in process managing and analysis are paramount.

**Four:** Clinical-reported outcomes measures (CROM) have to be normalized and standardized by international standards. Thus, the information of the clinical condition and the individual patient characteristics would be available for processing in real-time for both primary use (individual patient clinical management, economic evaluation, healthcare, quality, and safety management and studies) and secondary use (observational studies, clinical trials recruitment platforms, pragmatic clinical trials).

**Five:** The patient perspective is the core of the VBHC. Therefore, the role of the HIS for VBHC is paramount. Therefore, it is important to develop the PROM tools to evaluate the patients' and carers' quality of life and perceived quality. The main challenge for systematic data recording on health outcomes is to assure the baseline and follow-up of PROM and PREM since they cannot be recovered retrospectively by data managing or mining as the CROM can (if they have been appropriately recorded).

**Six:** The continuous improvement of the clinical conditions care process is the main objective of the VBHC framework. The PREM tools to evaluate the patient's subjective experience along the lifespan of care provided a fast and appropriate identification of critical improvement areas. Thus, we always accompany the VBHC implementation with PREM development. In parallel develop a professional-reported experience tool to evaluate the experience along their professional life should strengthen the project.

**Seven:** VBHC focus on real shared clinical and healthcare decision-making with a particular focus on the burden of treatment and the patient care plans. Developing the tools to analyze and visualize real-world data in real-time inpatient care is the leading resource for informed decision-making.

**Eight:** Another main objective of the VBHC is to establish a community of hospitals for best practices sharing and benchmarking, bearing in mind that it is not possible to adopt without an adaptation to the particular context of each institution.

**Nine:** We have also learned that data is, basically, imperfect and introduces bias in medical information. Thus, for data quality sake, the primary source should be the origin of the data in the system. Clinical information should be introduced by the clinician responsible for the data generation. If the information comes from analytical equipment, it should be imported directly, avoiding human interaction with the data recording. When data has to come from patients (or patient family), such as subjective data as symptoms or experience, it should be introduced by patients to the system. That helps to reduce interpretation bias and improves data quality. It is the main advance introduced by PROM vs. traditional quality of life questionnaires.

**Ten:** In this process, especially during the first 6 to 12 months, someone has to be responsible for the cohort follow-up to increase the data collection, project coordination, and advocacy (19). These professionals need skills in the data life cycle, quality and safety, process analysis, and interpersonal communication. The professionals in the quality units, services or directions, usually have a high level of these skills.

It has been a bumpy road, but we have learned valuable lessons to implement similar projects along the way. To date, all our projects used the ICHOM datasets (20–23), including CROM and PROM. We have developed our own set of PREM. In conclusion, there is a need to reduce missing and unclear data in real life, ensure the relevant information recording systematically outcomes, and record data from the primary source (clinician, patient). Implementing innovations such as VBHC is not “free of charge.” On the contrary, essential implementation costs must be considered (24).

## Data Availability Statement

The original contributions presented in the study are included in the article/Supplementary Material, further inquiries can be directed to the corresponding author.

## Ethics Statement

The studies involving human participants were reviewed and approved by Comité de Ética en investigación (CEIm)-Hospital Universitario 12 de Octubre. Written informed consent for participation was not required for this study in accordance with the national legislation and the institutional requirements.

## Author Contributions

CV-R has contributed to the conceptualization, main methodology, data extraction, check spelling data curation, writing and editing the original draft preparation, investigation, and supervision. AG-C has contributed to the conceptualization, check spelling, data curation, writing, and editing the original draft preparation. PR-L has contributed to the conceptualization and methodology of the article. BB-P has contributed to the data curation and critical review of the article. All authors have contributed to reviewing. All authors contributed to the article and approved the submitted version.

## Funding

Part of the research foundation of this work has been funded by Farmaindustria, Novartis Oncology, Novartis diagnosis, and Roche Diagnostics.

## Conflict of Interest

The authors declare that the research was conducted in the absence of any commercial or financial relationships that could be construed as a potential conflict of interest.

## Publisher's Note

All claims expressed in this article are solely those of the authors and do not necessarily represent those of their affiliated organizations, or those of the publisher, the editors and the reviewers. Any product that may be evaluated in this article, or claim that may be made by its manufacturer, is not guaranteed or endorsed by the publisher.

## References

[1] EIT Health. Implementing Value-Based Healthcare in Europe: Handbook for Pioneers (Gregory Katz director). Munich: VBHC (2020).

[2] Kaplan RS Porter ME. How to solve the cost crisis in health care. Harv Bus Rev. (2011) 89:46–52. 21939127

[3] Grupo AMPHOS. Informe AMPHOS: Medir para mejorar. Madrid: SEDISA (2019). (AMPHOS). Report No.: 11. Available online at: https://sedisa.net/wp-content/uploads/2019/12/informe_de_AMPHOS-07-2.pdf (accessed December 2, 2021).

[4] Fundación Signo. More Health Value. Available online at: https://www.morehealthvalue.com/ (accessed December 2, 2021).

[5] European Commission. Defining Value in Value-Based Healthcare. Available online at: https://ec.europa.eu/health/sites/default/files/expert_panel/docs/024_defining-value-vbhc_en.pdf (accessed December 2, 2021).

[6] ICHOM. ICHOM_What Matters Most. (2018). Available online at: https://ichom.org/files/books/ICHOM_Book.pdf (accessed December 2, 2021).

[7] Porter ME. What is value in health care? N Engl J Med. (2010) 363:2477–81. 10.1056/NEJMp1011024 21142528

[8] Reitblat C Bain PA Porter ME Bernstein DN Feeley TW Graefen M. Value-based healthcare in urology: a collaborative review. Eur Urol May. (2021) 79:571–85. 10.1016/j.eururo.2020.12.008 33413970

[9] Gray M Lagerberg T Dombrádi V. Equity and Value in ≪Precision Medicine≫. New Bioeth Multidiscip J Biotechnol Body abril de. (2017) 23:87–94. 10.1080/20502877.2017.1314891 28517992

[10] Gray M. Value-based healthcare. BMJ. (2017) 356:j437. 10.1136/bmj.j437 28130219

[11] Jones S Barlow D Smith D Jani A Gray M. Personalised and population healthcare for higher value. J R Soc Med March de. (2018) 111:84–7. 10.1177/0141076818758845 29432707 PMC5846946

[12] Porter ME. A strategy for health care reform–toward a value-based system. N Engl J Med. (2009) 361:109–12. 10.1056/NEJMp0904131 19494209

[13] Bauer MS Kirchner J. Implementation science: What is it and why should I care? Psychiatry Res enero de. (2020) 283:112376. 10.1016/j.psychres.2019.04.025 31036287

[14] van Egdom LSE Lagendijk M van der Kemp MH van Dam JH Mureau MAM Hazelzet JA. Implementation of Value Based Breast Cancer Care. Eur J Surg Oncol. (2019) 45:1163–70. 10.1016/j.ejso.2019.01.007 30638807

[15] Dombrádi V Bíró K Jonitz G Gray M Jani A. Broadening the concept of patient safety culture through value-based healthcare. J Health Organ Manag. (2021) 35:541–9. 10.1108/JHOM-07-2020-0287 33645172

[16] Gray M. Population healthcare: the third dimension. J R Soc Med. (2017) 110:54–6. 10.1177/0141076817691024 28169584 PMC5305013

[17] Gray M. Population healthcare: a new clinical responsibility. J R Soc Med. (2016) 109:437–8. 10.1177/0141076816679770 27923894 PMC5154409

[18] Ackerman IN Cavka B Lippa J Bucknill A. The feasibility of implementing the ICHOM standard set for hip and knee osteoarthritis: a mixed-methods evaluation in public and private hospital settings. J Patient-Rep Outcomes. (2017) 2:32. 10.1186/s41687-018-0062-5 30148249 PMC6091617

[19] Votova K Laberge A-M Grimshaw JM Wilson B. Implementation science as a leadership capability to improve patient outcomes and value in healthcare. Healthc Manage Forum. (2019) 32:307–12. 10.1177/0840470419867427 31446791

[20] Ong WL Schouwenburg MG van Bommel ACM Stowell C Allison KH Benn KE. A standard set of value-based patient-centered outcomes for breast cancer: the international consortium for health outcomes measurement (ICHOM) initiative. JAMA. (2017) 3:677–85. 10.1001/jamaoncol.2016.4851 28033439

[21] Kim AH Roberts C Feagan BG Banerjee R Bemelman W Bodger K. Developing a standard set of patient-centred outcomes for inflammatory bowel disease-an international, cross-disciplinary consensus. J Crohns Colitis. (2018) 12:408–18. 10.1093/ecco-jcc/jjx161 29216349

[22] Rodrigues IA Sprinkhuizen SM Barthelmes D Blumenkranz M Cheung G Haller J. Defining a minimum set of standardized patient-centered outcome measures for macular degeneration. Am J Ophthalmol. (2016) 168:1–12. 10.1016/j.ajo.2016.04.012 27131774

[23] Mak KS van Bommel ACM Stowell C Abrahm JL Baker M Baldotto CS. Defining a standard set of patient-centered outcomes for lung cancer. Eur Respir J. (2016) 48:852–60. 10.1183/13993003.02049-2015 27390281 PMC5007221

[24] Wensing M. Implementation science in healthcare: introduction and perspective. Z Evidenz Fortbild Qual Im Gesundheitswesen. (2015) 109:97–102. 10.1016/j.zefq.2015.02.014 26028446

